# Histone deacetylases inhibitor chidamide synergizes with humanized PD1 antibody to enhance T-cell chemokine expression and augment Ifn-γ response in NK-T cell lymphoma

**DOI:** 10.1016/j.ebiom.2022.104420

**Published:** 2022-12-31

**Authors:** Tingyu Wen, Guangyi Sun, Wenxin Jiang, Xiaohui He, Yuankai Shi, Fei Ma, Peng Liu

**Affiliations:** a Department of Medical Oncology, National Cancer Center/National Clinical Research Center for Cancer/Cancer Hospital, Chinese Academy of Medical Sciences and Peking Union Medical College, Beijing, 100021, China; b Department of Radiation Oncology, National Cancer Center/National Clinical Research Center for Cancer/Cancer Hospital, Chinese Academy of Medical Sciences and Peking Union Medical College, Beijing, 100021, China

**Keywords:** Immunotherapy, Cancer treatment combinations, H3K27ac, Pharmacology

## Abstract

**Background:**

Whether immunotherapy combined with different histone deacetylases (HDAC) inhibitors in refractory or relapsed natural killer/T-cell lymphoma (NKTCL) is superior to each agent is still lacking in head-to-head clinical trials or preclinical evidence.

**Methods:**

NKTCL cell line xenograft models (CDX) in immunocompetent, human programmed cell death protein 1 (PD1) knock-in genetically engineered mice were used to investigate the combination effects. Different types and dosages of HDAC inhibitors were investigated. We explored the underlying mechanisms by RNA-sequencing and ChIP-sequencing. Two clinical cases treated with anti-PD1/chidamide were presented.

**Findings:**

Anti-PD1/chidamide shows significant tumour rejection in two CDX models. RNA-seq and CHIP-seq revealed that chidamide is synergistic to enhance T-cell chemokine expression, augment the Ifn-γ response, and increase CD8 T-cell infiltration via histone modification. Ifn-γ neutralizing antibody can attenuate the efficacy of combination drugs. However, the anti-PD1/romidepsin failed to augment the Ifn-γ response. The expressions of Ifn-γ related gene set signatures are significantly correlated with tumour rejection in anti-PD1/chidamide. In the clinic, two NKTCL patients treated with the PD1/chidamide show promising efficacy and limited toxicity.

**Interpretation:**

Anti-PD1/chidamide enhances T-cell chemokine expression and augments the IFN-γ response in preclinical NKTCL immunocompetent models. IFN-γ signatures may be good response biomarkers for the selection of potentially benefit patients.

**Funding:**

This study was supported by the Chinese National Major Project for New Drug Innovation (2017ZX09304015) and the 10.13039/501100009812Chinese Society of Clinical Oncology Research Fund (Y-BMS2019-026).

**Research in context**

**Evidence before this study**

R/R NKTCL lacks standard care and requires more effective new drugs or combinations. A phase 2 clinical trial led by our group suggested that chidamide, an oral selective inhibitor of histone deacetylase, responds to a part of R/R NKTCL. The NCCN guidelines also suggest HDAC inhibitor and anti-PD1 may be salvage options for R/R NKTCL. However, whether immunotherapy combined with chidamide in R/R NKTCL is superior to each agent is still lacking in head-to-head clinical trials or preclinical evidence.

**Added value of this study**

Different histone deacetylase inhibitors combined with immunotherapy modulate different antitumor immunity in the human PD1 gene knock-in immunocompetent mice model. Chidamide synergizes with checkpoint inhibition by enhancing T-cell chemokine expression and augmenting the Ifn-γ response, but the Romidepsin/anti-PD1 combination didn't show significant changes in our study. Ifn-γ related signatures may be good predictive biomarkers for the selection of potentially benefit patients. The chemo-free regimen of chidamide combined with anti-PD1 treatment showed promising efficacy and mild toxicity in clinical cases.

**Implications of all the available evidence**

Our findings suggest that anti-PD1/chidamide is one of the promising chemo-free regimens for R/R NKTCL, especially for older and fragile patients. A clinical trial assessing anti-PD1/chidamide is the warrant, and Ifn-γ related signatures may be good predictive biomarkers.

## Introduction

Natural killer/T-cell lymphoma (NKTCL) is a kind of non-Hodgkin lymphoma (NHL) derived from mature natural killer/T cells. The incidence of NKTCL is more frequent in East Asia and Latin America, with an obvious regional distribution difference.^1^^,^^2^ Although NKTCL localized to the nasal region is responsive, patients with refractory or relapsed (R/R) NKTCL have a poor prognosis after failing asparaginase-based first-line treatment, and there is a lack of effective standard options. The 5-year overall survival rate in the era of l-asparaginase is still less than 40%.^3^^,^^4^ Optional therapies for R/R NKTCL are limited and most options are intolerable for elder or fragile patients. Combination chemotherapy regimens, such as DHAP (dexamethasone-cytarabine-cisplatin), ICE (ifosfamide-carboplatin-etoposide), GDP (gemcitabine-dexamethasone-cisplatin), dexamethasone-cisplatin-gemcitabine-asparaginase) and P-Gemox (asparaginase-gemcitabine-oxaliplatin), are the main options. However, the median age of NKTCL is approximately 44 years,^4^ and patients are usually older and in a frail state after a first-line combination chemotherapy regimen. More effective new drugs and combinations, especially chemo-free regimens, are required.

Recently, the emergence of programmed death-1 (PD1) and programmed death ligand-1 (PD-L1) monoclonal antibodies has changed the therapeutic patterns of various tumours. Both PD1/PD-L1 antibodies showed promising efficacy in Hodgkin lymphoma and some solid tumours, but their clinical application in NKTCL needs to be further explored. Preclinical studies have suggested that T-cell lymphoma has a higher expression rate of PD-L1 than B-cell lymphoma, and the expression rates of PD-L1 in extranodal NKTCL and peripheral T-cell lymphoma, not otherwise specified (PTCL-NOS), were 39% and 26%, respectively.^5^ Clinical trials showed that the objective remission rate of PD1 antibody monotherapy for R/R NKTCL was 57.1–67.9% and the 2-year overall survival rate was 78.6%.^6^, ^7^, ^8^ Another study showed that the objective remission rate of avelumab (PD-L1 antibody) for R/R NKTCL was 38%, while the complete remission rate was 24%.^9^ This suggests that the PD1/PD-L1 antibody may be beneficial for NTKCL treatment. However, the selection of potentially benefit patients and improved limited efficacy may be unmet clinical needs.

On the other hand, the histone deacetylase (HDAC) inhibitors belinostat and romidepsin are recommended in the National Comprehensive Cancer Network guidelines (version 2.2022) for R/R extranodal NKTCL, but the strength of evidence is weak and should be used in certain circumstances. Chidamide, another selective inhibitor of HDAC1, 2, 3, and 10 from China, was approved for R/R peripheral T-cell lymphoma by the China Food and Drug Administration based on our group lead phase II clinical trial.^10^ The overall response rate (ORR) of chidamide in angioimmunoblastic T-cell lymphoma (which is characterized by numerous epigenetic mutations), PTCL-NOS, and NKTCL is 50%, 22%, and 19%, respectively.^10^ Other HDAC inhibitors, belinostat and romidepsin, have shown similar efficacy in NKTCL.^11^^,^^12^ HDAC inhibitors are chemo-free options for R/R NKTCL, with limited efficacy.

Several pre-clinical studies have reported that HDAC inhibitors favour immunotherapy response in solid tumours; however, clinical trials showed limited efficacy and most trials were suspended.^13^ The reason for the failure from basic to clinical translation may be the lack of scientific pre-clinical models for pharmacodynamic evaluation. Most of these pre-clinical studies used murine PD1 antibodies tested in one type of murine cell line-derived xenograft (CDX). However, murine PD1 antibodies with different clones (RMP1-14, 29 F.1A12, J43 et al.) may have different effects. The ideal model for immunotherapy may be as follows: 1) immunocompetent with a fully functional immune system; 2) immune cells expressing human PD1 protein that can directly work with commercial humanized PD1 antibodies. Humanized PD1 mice that knock the human PD1 extracellular segment into the mouse PD1 transcription initiation site (ATG) position did not change the segment of the transmembrane and cytoplasmic regions, which may meet the above requirements for preclinical immunotherapy combination testing.^14^ No signal transduction or immune microenvironment changes were observed between knock-in and wild-type mice. Notably, no immunocompetent models testing humanized PD1 antibody with HDAC inhibitors in NKTCL have been reported.

Although a single-arm clinical trial reported that PD1 antibody has promising efficiency in NKTCL, there is no report of a randomized, head-to-head clinical trial or preclinical animal study to explore whether PD-1 antibody combined with a histone deacetylase inhibitor is superior to every single agent and shows synergistic. Therefore, this pre-clinical study aimed to investigate the combined effect of the humanized PD1 antibody and different HDAC inhibitors in human PD1 gene knock-in mouse models, illustrate the underlying mechanisms, and discover predictive biomarkers.

## Methods

### Cell lines and mice

EL4 and RMA cell lines were purchased from the Chinese National Infrastructure of Cell Line Resources and authenticated with STR profiling. EL4 and RMA cell lines are PDL1-expressed NKTCL cell lines that are induced by 9,10-dimethyl-1,2-benzoanthracene in C57BL/6 N mice.^15^, ^16^, ^17^ C57BL/6-Pdcd1^em1 (hpdcd1)/Smoc^ of 6-8-week-old male humanized PD1 (hPD1) mice were purchased from Shanghai Model Organisms Center, Inc. Institutional animal ethics committee of Cancer Hospital Chinese Academy of Medical Science (NCC2020A121) was approved for the use of mice in this study.

### Reagents

RPMI-1640, 0.25% trypsin, PBS, and fetal bovine serum were purchased from Thermo Fisher Scientific, Inc. (San Diego, CA, USA). The humanized PD1 antibody tislelizumab was purchased from Beijing Gene Co., Ltd. (Beijing, China). The HDAC inhibitor, chidamide and romidepsin, was purchased from MCE Chemexpress Co., Ltd. (HY-15149, HY-109015, Shanghai, China). Erythrocyte lysates were purchased from Sangon Biotech Co., Ltd. (B541001, Shanghai, China).

### Mononuclear cell surface staining with tislelizumab

After 6–8 weeks, the spleen from hPD-1 transgenic mice was separated, ground into a cell suspension in a culture dish, and filtered through a stainless-steel 400 mesh. The cell suspension was then centrifuged for 5 min at 1500r/min. Red blood cells were removed by incubation with the Tris-NH_4_Cl erythrocyte lysate. The mononuclear cells were resuspended and incubated in RPMI-1640 medium containing 10% fetal bovine serum and activated by adding 2 μg/mL CD3 and CD28 antibodies and 25 μg/mL IL2. After 72 h, the cells grew into clusters and were successfully activated. The activated mononuclear cells were re-suspended in 100 μL PBS containing 5 μL 200 μg/mL tislelizumab, incubated at 4 °C for 20 min. The cells were then washed twice with PBS and added 5 μL mouse anti-human IgG Fc antibody (mouse IgG2a, κ, Cat. #: 409304, BioLegend, 200 μg/mL), incubated for 20 min washed twice, re-suspended in 500 μL of PBS, and subjected to flow cytometry.

### Murine syngeneic tumour model

EL4 and RMA cells were cultured in RPMI-1640 medium containing 10% fetal bovine serum at 37 °C and 5% CO2. The culture medium was changed before inoculation. The amounts of mice are determined by the resource equation method. E = N−B−T, where E is the error df and should be between 10 and 20, N is the total df, B is the blocks df, and T is the treatments df. In a non-blocked design, the equation reduces to E = N−T should be 10–20. At the same time, we must also consider that there are enough materials for various experiments. On the day of inoculation, the right flank of hPD1 mice was inoculated with a total of 1 × 10^6^ EL4 or RMA cells. After the tumour reached approximately 80–150 mm^3^, mice were randomized and treatments were administered as shown in the scheme. Tumour volume and mouse weight were measured every three days. The tumour growth inhibition rate was calculated as follows: (1-tumour volume of the experimental group)/(tumour volume of the vehicle control group). Tumour volume was calculated as 1/2 × (width^2^ × length). When the maximum diameter exceeded 20 mm, the mice were sacrificed and tumours were collected. Blood tests to predict hematological toxicity were performed using the IDEXX ProCyte. Bliss independence is described by the equation EAB = EA + EB − EA × EB to define the synergistic, additive, and antagonistic effects.^18^ EAB is the ratio of the 2-drug combination group to the control group, and EA or EB is the ratio of the single-drug group to the control group. If the EAB is greater than the calculated score, this suggests a synergistic effect. If the EAB is approximate to the calculated score, it suggests an additive effect. If EAB is less than the calculated score, it suggests an antagonistic effect.

### RNA sequencing and data analysis

RNA sequencing of tumours was performed using the Illumina HiSeq next-generation sequencing system as previously reported.^19^ Absolute gene expression was quantified using the number of reads per million (TPM). Differentially expressed genes (DEG) were evaluated using DESeq and displayed as log 2-fold change (FC) with adjusted *P* values for multiple testing.^20^ GO/KEGG and Gene set enrichment analysis (GSEA) was performed with normalized expression data.^21^ Ingenuity pathway analysis (IPA) of upstream regulators, canonical pathways, and diseases and functions was performed as previously reported.^22^ The string protein–protein interaction network was used to identify hub genes.^23^ The immune cell abundance identifier for mouse (ImmuCellAI-mouse) is a tool developed to infer the relative proportion of immune cells from mouse transcriptome data.^24^ Signature scores were calculated using the mean log2 (TPM including genes)-log2 (TPM housekeeping genes).^25^^,^^26^ RBA-seq data were deposited in the NCBI database under the accession number PRJNA901065.

### Western blot

Tumours from each group were minced and lysed in lysis buffer for immunoblotting (P0013, Beyotime biotechnology) with deacetylase inhibitor cocktail (P1113, Beyotime biotechnology). Proteins were separated in SDS polyacrylamide gel, electro-transferred onto nitrocellulose membranes (FFN08, Beyotime biotechnology), and blocked-in blocking buffer (P0023B, Beyotime biotechnology). Then, the membranes were incubated with different antibodies overnight: Ifn-γ (15365-1-AP, Proteintech, 1:2000 dilution), Cxcl9 (701117, Invitrogen, 1:500 dilution), Cxcr3 (ab288437, Abcam, 1:1000 dilution), Acetyl-histone H3 (8173 T, CST, 1:1000 dilution), Histone H3 (4499 T, CST, 1:3000 dilution), Pdl-1 (66248-1-Ag, Proteintech, 1:5000 dilution), β-actin (4970 T, CST, 1:1000 dilution). Corresponding secondary antibodies at room temperature for 2 h. The protein bands were visualized in Imagequant 800 system.

### Enzyme-linked immunosorbent assay (ELISA)

Proteins were extracted from tumours as the methods in western blot. Tissue homogenates were detected in the Cxcl9 ELISA kit (CSB-EL006252MO, Cusabio) and Ifn-γ ELISA kit (KE10001, Proteintech). These assays use the sandwich enzyme immunoassay technique in combination with the enzyme–substrate chromogenic reaction to quantify the analyte in the samples. The colour develops positively to the amount of Cxcl9 or Ifn-γ in samples. The colour intensity is measured at 450 nm via the H1 Biotek microplate reader.

### Chromatin immunoprecipitation sequencing (ChIP-seq) and CHIP-qPCR

ChIP assays were performed using SimpleChIP® Plus Enzymatic Chromatin IP Kit (Magnetic Beads, #9005, CST), as per the manufacturer's protocol. Fragmented chromatin was immunoprecipitated with anti-H3K27ac (8173 T, CST) and negative control rabbit IgG. The purified DNA fragments were subjected to ChIP-seq and validated in quantitative PCR. The ChIP-qPCR results are expressed as enrichment relative to input. ChIP-seq libraries were constructed using the VAHTS Universal DNA Library Prep Kit for Illumina V3 (ND607, Vazyme). The library products were sequenced on Novaseq 6000 sequencer (Illumina). ChIP-seq data were deposited in the NCBI database under the accession number PRJNA901065. ChIP-qPCR primers are as follows: Ifnγ-1 5′- AGTTCTGGGCTTCTCCTCCT-3′, 5′-ACAGCCAGAAACAGCCATGA-3’; Ifn-γ2 5′-ACATTAAGAACTTTGCCTCCCA-3′, 5′- TCAGACGTCTTGAAAGTGATAGTGA-3’; Ifn-γ3 5′-TGGTTCTTATTCCTTCAGACGTC-3′, 5′- TTCCCTGCGTAGTTTTGGTG-3’; Cxcr3-1 5′- ATTCGATGCTGCTCACTCAG-3′, 5′- TACAATACAGCGGCCAAGTG-3’; Cxcr3-2 5′- CTGCATTCGATGCTGCTCAC-3′, 5′- TAAGTTCTGCTGCCCAGCTC-3’.

### Immunohistochemistry (IHC)

The dissected tumours were dewaxed and hydrated, after blocking the non-specific sites by 5% BSA, the tissues were incubated with primary antibody DSN1 overnight at 4 °C. After washing the tissues were overlaid with appropriate secondary antibody for 2 h at room temperature, then detected using DAB reagent.

The tumours were fixed in 4% paraformaldehyde and cut into 4-μm sections. Before staining, all sections were subjected to antigen retrieval and blocking of endogenous peroxidase. Slides were incubated with the primary antibodies Ifn-γ (15365-1-AP, Proteintech, 1:200 dilution), Cd8 (ab217344, Abcam, 1:2000 dilution), Pdl-1 (66248-1-Ag, Proteintech, 1:10000 dilution), Cxcl9 (701117, Invitrogen, 1:200 dilution) overnight, and the rabbit or mouse secondary antibody at room temperature for 1 h. DAB was added to visualize the antigens in situ. The hematoxylin and eosin staining was performed according to the established protocols.^25^

### Clinical data collection

Clinical cases of NKTCL patients treated with chidamide and PD1 antibody were collected from the Cancer Hospital, Chinese Academy of Medical Sciences, and Peking Union Medical College. The collection of clinical cases was approved by the Ethics Committee of Cancer Hospital, Chinese Academy of Medical Sciences, and Peking Union Medical College (No. 19-018/1803). Written informed consent was obtained from all the patients.

### Statistical analysis

Continuous variables are plotted as mean ± SD or median (interquartile range) as indicated. The assumption of normality was checked by the Anderson-Darling test or the Shapiro–Wilk test. Statistical significance was determined using Student's t-test, Mann–Whitney U-test, one-way analysis of variance (ANOVA), Spearman correlation analysis, and two-way ANOVA using GraphPad Prism software version 7.0. Statistical significance was set at ∗P < 0 0.05, ∗∗P < 0.01, and ∗∗∗P < 0.001, respectively.

### Role of the funding source

The funding sources were not involved in the study design; data collection, analysis, and interpretation of data; or decision to publish.

## Results

### Romidepsin combined with hPD1 antibody is superior to PD1 antibody alone in the treatment of NK-T cell lymphoma, but it is similar to romidepsin single agent, either in different dosages or sequence

To determine whether the humanized PD1 antibody tislelizumab could bind to immune cells of the hPD1 mice model, the PE anti-human IgG FC secondary antibody was labelled after tislelizumab was incubated with mononuclear cells. Flow cytometry showed that the humanized PD1 antibody could efficiently bind to mononuclear cells (Supplementary Fig. S1c). Moreover, RNA sequencing of tumours isolated from CDXs also proved that CDXs display mosaic expression of molecules of NK-T origin (Supplementary Fig. S1a, b).

In the preliminary experiment, we used a small sample size (n = 4) to explore the efficacy of romidepsin combined with PD1 monoclonal antibody under different medication sequences or dosage conditions (data not presented). To compare the efficacy of low-dose (1/3 maximum tolerance dosage, MTD) romidepsin sequentially or simultaneously combined with the PD1 antibody, sequential administration was performed two days after the administration of romidepsin. The tumour growth curve showed that the simultaneous administration group and sequential administration group had significantly inhibited tumour growth compared with PD1 monotherapy. However, there was no difference between the simultaneous and sequential administration groups. The simultaneous administration group and sequential administration group showed inhibited tumour growth compared with the romidepsin monotherapy group, with no statistical difference. When comparing the efficacy of high-dose romidepsin sequentially or simultaneously with PD1 antibody, high-dose romidepsin significantly inhibited tumour growth compared with PD-1 monotherapy, either in the simultaneous administration group or the sequential administration group. However, there was no significant difference between the simultaneous and sequential administration groups. The simultaneous and sequential administration groups were not superior to PD1 monotherapy. When comparing the efficacy of low-dose or high-dose romidepsin simultaneous with PD1 antibody, there was no difference between the high-dose and low-dose combination groups. High-dose romidepsin, low-dose romidepsin, high-dose combination, and low-dose combination inhibited tumour growth compared with PD1 monotherapy. However, the high-dose combination showed a similar response as high-dose romidepsin monotherapy. The low-dose combination inhibited tumour growth compared with low-dose romidepsin monotherapy, with no significant difference. When comparing the efficacy of low-dose or high-dose romidepsin sequentially with PD1 antibody, there was no difference between the high-dose sequential group and the low-dose sequential group. Similarly, high-dose romidepsin, low-dose romidepsin, high-dose sequential group, and low-dose sequential group inhibited tumour growth compared with PD1 monotherapy. The high-dose sequential group and high-dose romidepsin monotherapy showed similar efficacies. The low-dose sequential group showed inhibition compared to the low-dose romidepsin monotherapy group, with no significance. All mice survived during drug administration. No significant changes in body weight were observed among the groups.

**Fig. 1:**
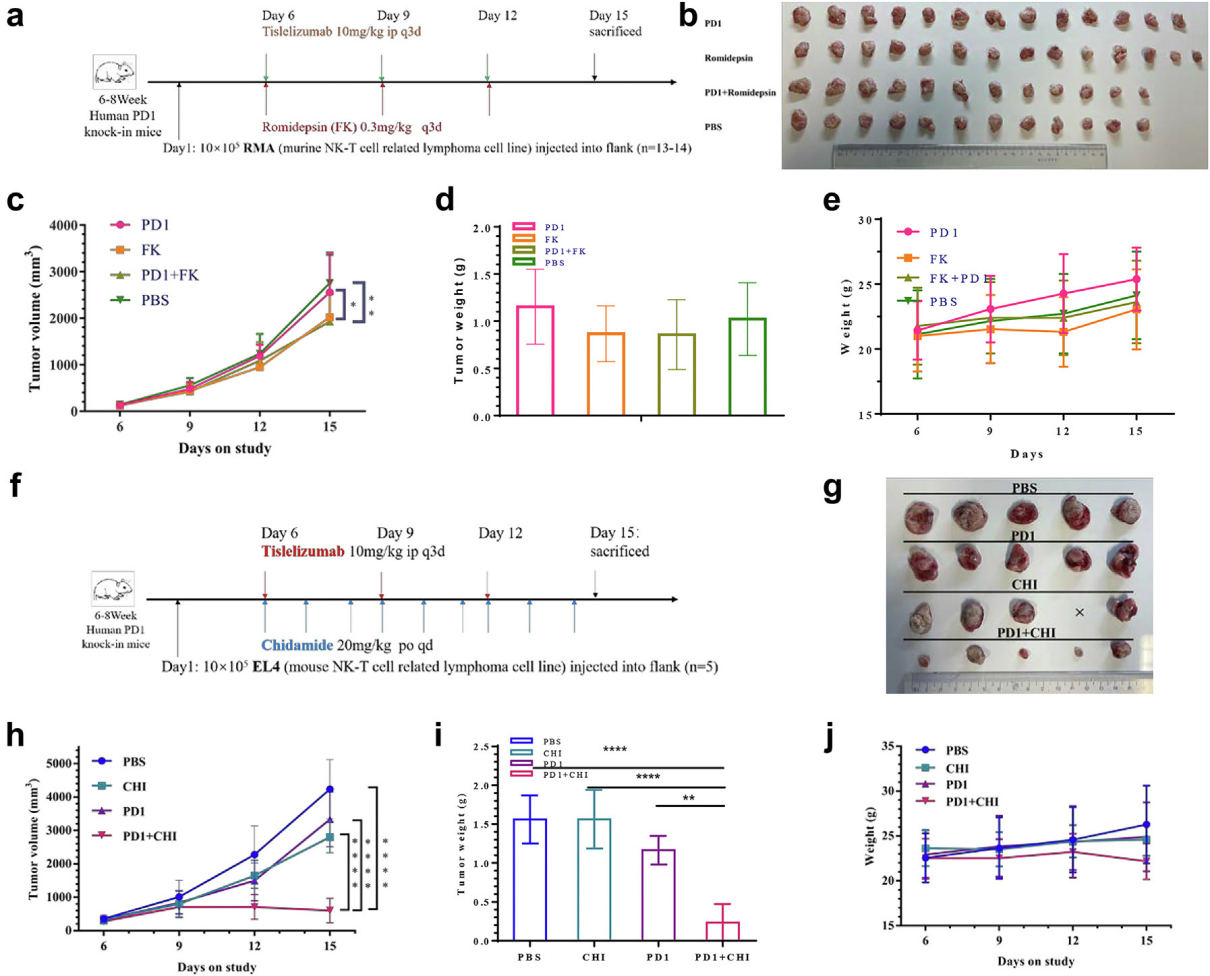
Different HDAC inhibitors combined with humanized PD1 antibody in immunocompetent NK-T lymphoma CDX. **a**, Treatment scheme. Human PD1 knock-in mice bearing RMA tumours were treated starting 6 days after tumour implantation with romidepsin (FK; 0.3 mg/kg q3d i.p.) and/or anti-PD1 (PD10; 10 mg/kg q3d i. p.) concurrently. **b**, Tumour specimens after treatment. **c**, Tumour volume, **d**, tumour weight and **e**, mice weight changes during treatment. Bliss independence model suggests the combination of romidepsin and PD1 antibody exist an additive effect. **f,** Treatment scheme. Human PD1 knock-in mice bearing EL4 tumours were treated starting 6 days after tumour implantation with chidamide (CHI; 20 mg/kg QD oral) and/or anti-PD1 (PD1; 10 mg/kg q3d i. p.). **g,** Tumour specimens after treatment. **h,** Tumour growth curve for chidamide combined with PD1 antibody. **i,** Tumour weight and **j,** mice weight changes in each group. Bliss's independence model suggests the combination exist a synergistic effect (∗*P*＜0.05，∗∗*P*＜0.001，∗∗∗*P*＜0.0001，∗∗∗∗*P*＜0.00001, Mann–Whitney U-test, one-way ANOVA and two-way ANOVA).

**Supplementary Fig. S2:**
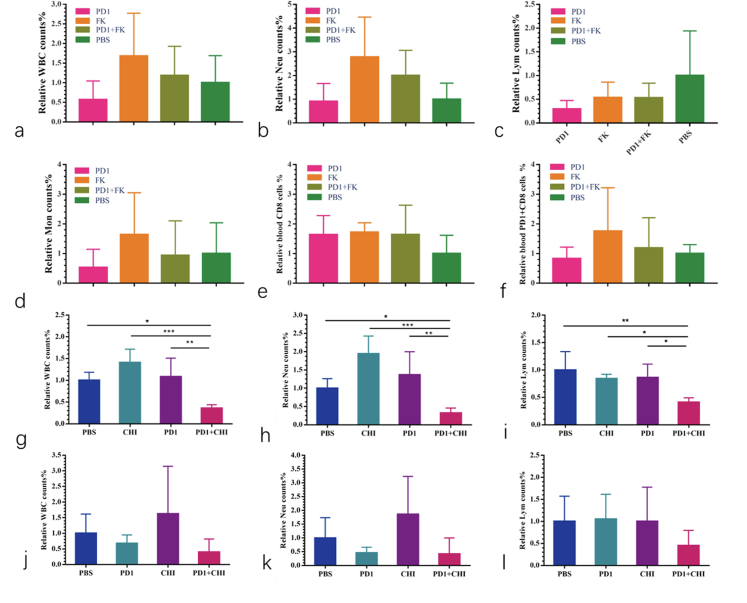


Subsequently, we verified the efficacy of the low-dose concurrent combination in another NKTCL CDX model, in the larger sample size cohort (Fig. 1a). Unsurprisingly, romidepsin combined with PD1 antibody or romidepsin monotherapy was superior to PD1 antibody monotherapy or vehicle in the treatment of NK-T cell lymphoma, but the combination was not superior to romidepsin alone. Changes in tumour weight or mouse weight were not different between the combination and monotherapy groups. One mouse died in each group (PD1 monotherapy, combination, and vehicle groups) on day 17. The tumour inhibition rates of PD1, romidepsin, and their combination were 7.2%, 26.6%, and 30.1%, respectively. The bliss independence model suggests that the combination of romidepsin and PD1 antibody has an additive effect, at least not a synergistic effect. Hematologic toxicity in the combination group was mild, and it may have caused a slight increase in PD1-positive CD8 T cells (Supplementary Fig. S2a–f).

### Combination of chidamide and humanized PD1 antibody enables tumour rejection in two immunocompetent syngeneic tumour models

Although romidepsin and PD1 antibodies show no synergistic effect, the existence of other HDAC inhibitors is not well known. Therefore, we tested the efficacy of another HDAC inhibitor, chidamide, combined with a humanized PD1 antibody, in these two CDX models. For EL4 CDX, chidamide was orally administered once a day. The tumour growth curve showed that the combination group was superior to the PD1 monotherapy, chidamide monotherapy, and vehicle groups. Chidamide or PD1 monotherapy was superior to the vehicle group. Chidamide showed greater suppression of tumour growth than PD1 monotherapy, with no statistical difference (Fig. 1f–h). The tumour inhibition rates of PD1, chidamide, and their combination were 21.3%, 33.9%, and 85.7%, respectively. The bliss independence model suggests that the combination of chidamide and PD1 antibody has a strong synergistic effect. Tumour weight changes in the combination group were significantly higher than those in the PD1 antibody, chidamide, and vehicle groups (Fig. 1i). The weight changes in each group were not significant (Fig. 1j). Blood tests indicated acceptable toxicity (Supplementary Fig. S2g–i).

**Fig. 2:**
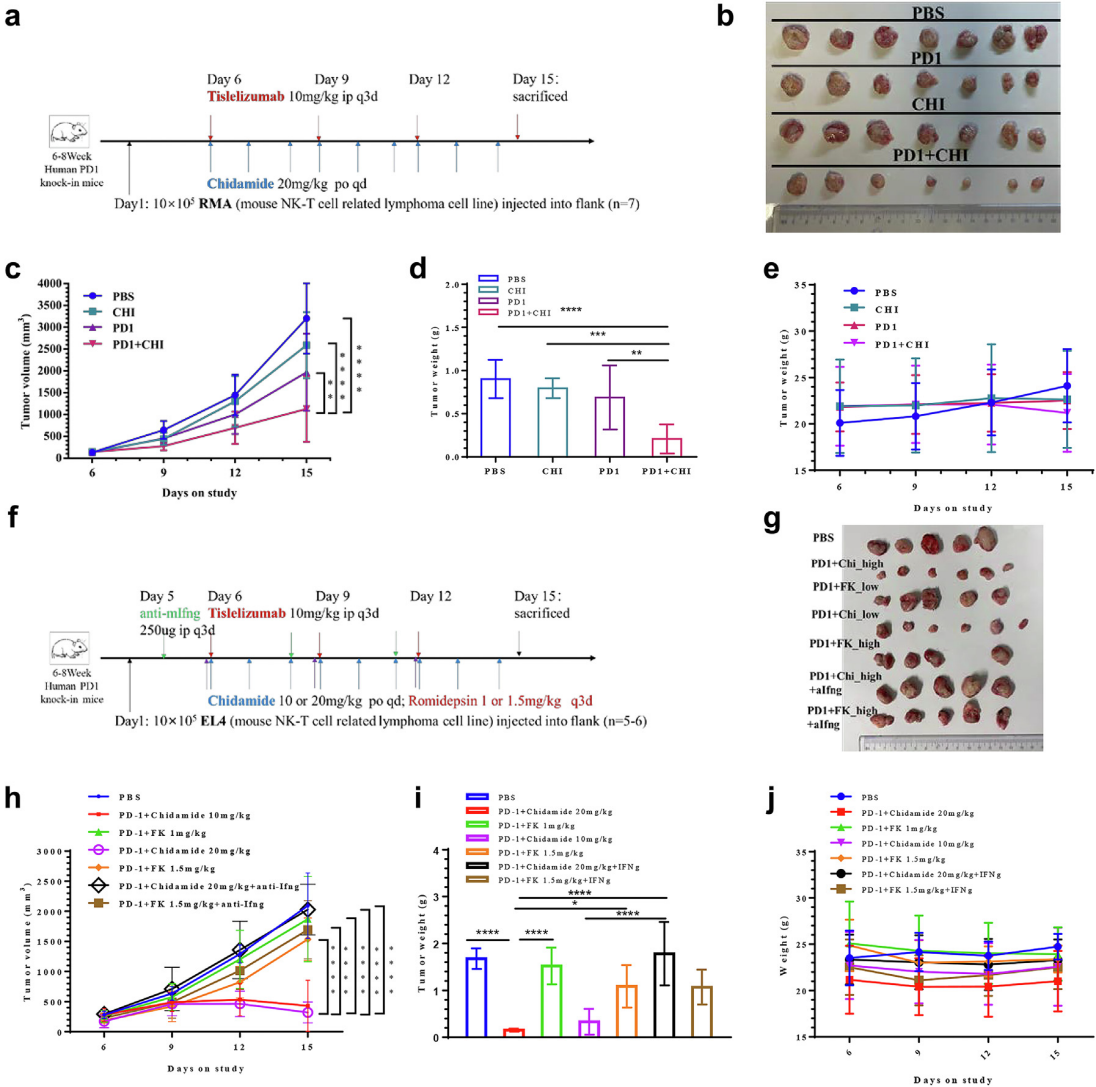
Anti-PD1/chidamide enables tumour rejection and is dependent on Ifn-γ. **a**, Treatment scheme. Human PD1 knock-in mice bearing RMA tumours were treated starting 6 days after tumour implantation with chidamide (CHI; 20 mg/kg QD oral) and/or anti-PD1 (PD1; 10 mg/kg q3d i. p.) concurrently. **b**, Tumour specimens after treatment. **c**, Tumour volume, **d**, tumour weight and **e**, mice weight changes during treatment. Bliss independence analysis also suggests the combination exist a synergistic effect. **f,** Treatment scheme. Human PD1 knock-in mice bearing EL4 tumours were treated starting 5 days after tumour implantation with or without anti-Ifn-γ antibody. **g,** Tumour specimens after treatment. **h**, growth curve. **i,** Tumour weight. **j,** Mice weight changes (∗*P*＜0.05，∗∗*P*＜0.001，∗∗∗*P*＜0.0001，∗∗∗∗*P*＜0.00001, Mann–Whitney U-test, one-way ANOVA and two-way ANOVA).

In RMA CDX, the tumour growth curve also showed that the combination group was superior to the PD1 monotherapy, chidamide monotherapy, and vehicle groups. PD1 monotherapy was superior to that in the vehicle group. Chidamide showed greater suppression of tumour growth than PD1 monotherapy or the vehicle group, with no statistical difference (Fig. 2a–c). The tumour inhibition rates of PD1, chidamide, and their combination were 38.5%, 19.1%, and 64.6%, respectively. The bliss independence model suggests that the combination of chidamide and PD1 antibody has a strong synergistic effect. Tumour weight changes in the combination group were significantly greater than those in the PD1 antibody, chidamide, and vehicle groups (Fig. 2d). Changes in mouse weight in each group were not obvious (Fig. 2e). Blood tests indicated acceptable blood toxicity (Supplementary Fig. S2 j–l).

### Chidamide synergizes with humanized PD1 antibody to enhance T-cell chemokine expression and augment IFN-γ response in pre-clinical NKTCL model

**Fig. 3:**
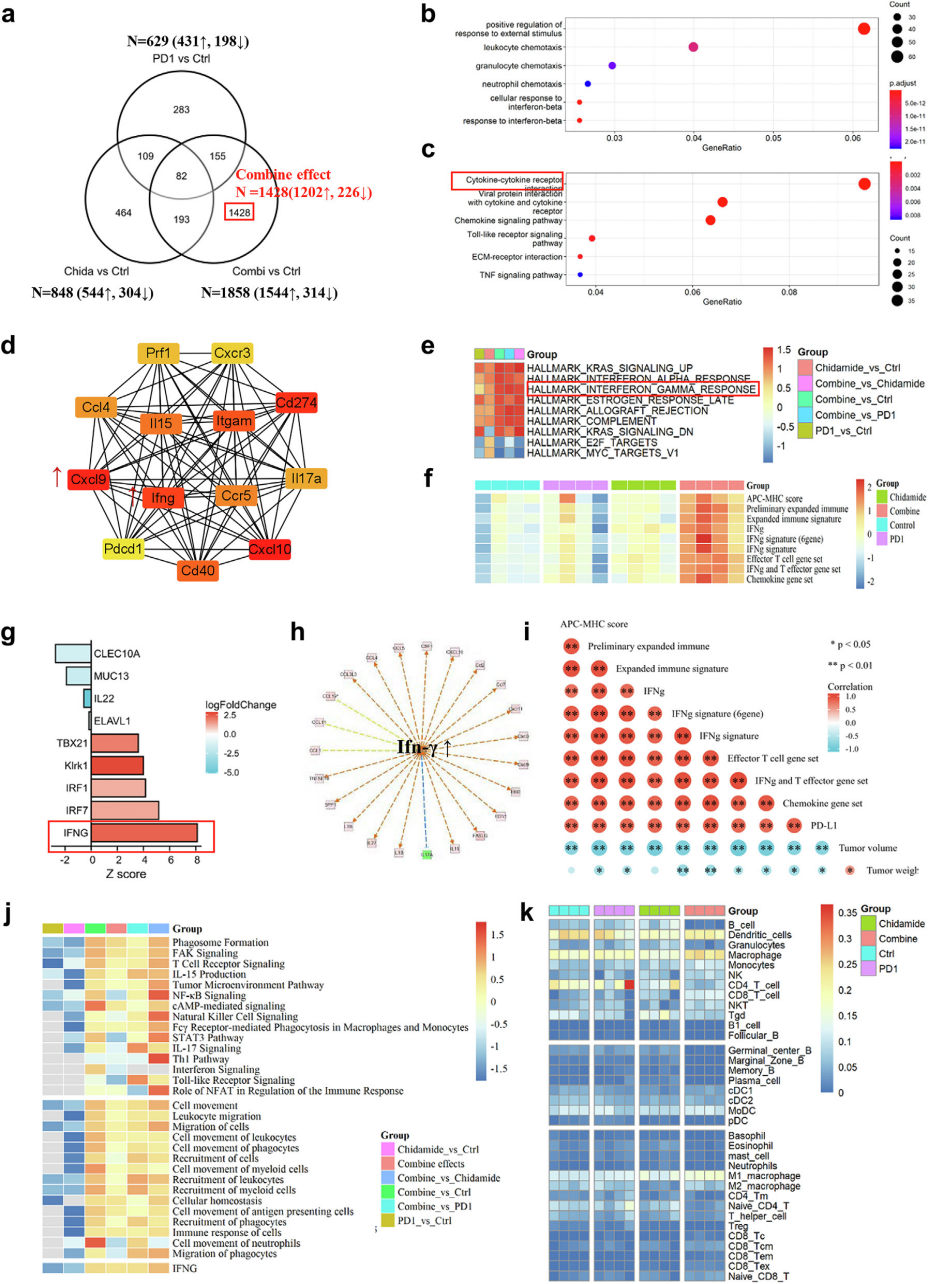
The combination of chidamide and humanized PD1 antibody markedly enhances T-cell chemokine expression and augment IFN-γ response in the pre-clinical NKTCL model. **a**, Summary of shared and nonoverlapping DEGs with several upregulated and downregulated genes across treatment groups is shown. 1428 genes which only regulated in combination groups are definite as the combined effect. **b**, Go analysis of upregulated genes in combined effect. **c**, KEGG analysis of upregulated genes in combined effect. **d**, String protein–protein analysis of combined effect to explore hub genes in the regulated network. **e**, Heatmap of GSEA analysis in different comparisons. **f**, Heatmap of gene set expression scores in different groups. **g**, Ingenuity pathway analysis predicts hub upstream regulator in the combined effect. **h**, downstream genes which may be regulated by IFN-γ. **i**, the correlation analysis of each score and tumour volume or weight. **j**, Ingenuity pathway analysis of canonical pathway (Upper) and diseases & function (Below) changes in each comparison. **k**, Immune cell proportion estimated based on RNA-seq data (∗*P*＜0.05，∗∗*P*＜0.001, Spearman correlation analysis).

Different HDAC inhibitors have different effects when combined with immunotherapy. We then performed RNA-seq of the tumour tissues to elucidate the underlying mechanism. A single agent PD1 antibody or chidamide modulated the expression of several genes (629 and 848 DEG, respectively). Moreover, the combination yielded prominent gene expression changes, and 1428 (1428/1858) non-overlapping DEG were uniquely modulated by the combination treatment (Fig. 3a, Supplementary Table S1). GO/KEGG cluster analysis of DEG in the combination group showed that the combination treatment significantly upregulated genes involved in immune-related pathways, such as immune cell chemotaxis, cytokine interaction, and TNF responses (Fig. 3b and c). To investigate the hub genes in the combination group, string protein–protein interaction network analysis suggested that the hub genes were Cxcl9, Cxcl10, IFN-γ, and CD274 (Fig. 3d). IPA upstream analysis also suggested that IFN-γ is the main upstream regulator (Supplementary Table S2) and can regulate several downstream genes which are known as immune activation, especially several cytokine-and chemokine-regulated genes (Fig. 3g, h, Supplementary Table S2).

To compare immune-related molecular and cellular pathway changes in each group, GSEA hallmark analysis in pairwise comparisons was performed. Interferon alpha and gamma response hallmarks were significantly upregulated in the combination group compared to those in the monotherapy group (Fig. 3e). IPA comparison analysis in canonical pathways suggests that immune pathways, such as T cell receptor signalling, tumour microenvironment pathway, natural killer cell signalling, and the stat 3 pathway, are all activated in the combination therapy (Fig. 3j). IPA comparison analysis of diseases and biological functions also suggested that immune cell recruitment, movement, and migration were activated by the combination treatment (Fig. 3j).

Immune cell composition is an important factor that determines the efficacy of anti-tumour treatment. ImmunceCellAI-mouse estimated the abundance of immune cells, indicating that B cells, germinal centre B cells, and marginal zone B cells decreased in the combination, and the abundance of M1 macrophages, cytotoxic CD8 T cells, and CD8 effector memory T cells increased in the combination (Fig. 3k).

Previously, RNA-seq data suggested that combination treatment induces the secretion of chemokines and cytokines, and whether chemokine or cytokine mRNA profiles can be useful in predicting the response to PD-L1 checkpoint blockade is unknown. Therefore, we explored the correlation between tumour volume and the expression score of different gene sets, including Pdl1, IFN-γ, IFN-γ signature (6-gene), IFN-γ signature (10-gene), antigen-processing machinery and major histocompatibility complex class I and II gene expression (APC-MHC) score (15-gene), preliminary expanded immune (28-gene), expanded immune signature (18-gene), effector T cell gene set (6-gene), IFN-γ and T effector gene set (8-gene), and chemokine gene set (9-gene). Gene set scores in the combination treatment were significantly over-expressed (Fig. 3I–L, Supplementary Fig. S3). Tumour volume and weight were significantly negatively correlated with each score (Fig. 3f).

Collectively, these data demonstrate the development of an integrated antitumor immune response mediated by a combination of chidamide/anti-PD1 and suggest that the underlying mechanism involves immune cell trafficking, infiltration, and killing of cancer cells.

### PD1 antibody combined with romidepsin shows no significant changes in immune responses

**Fig. 4:**
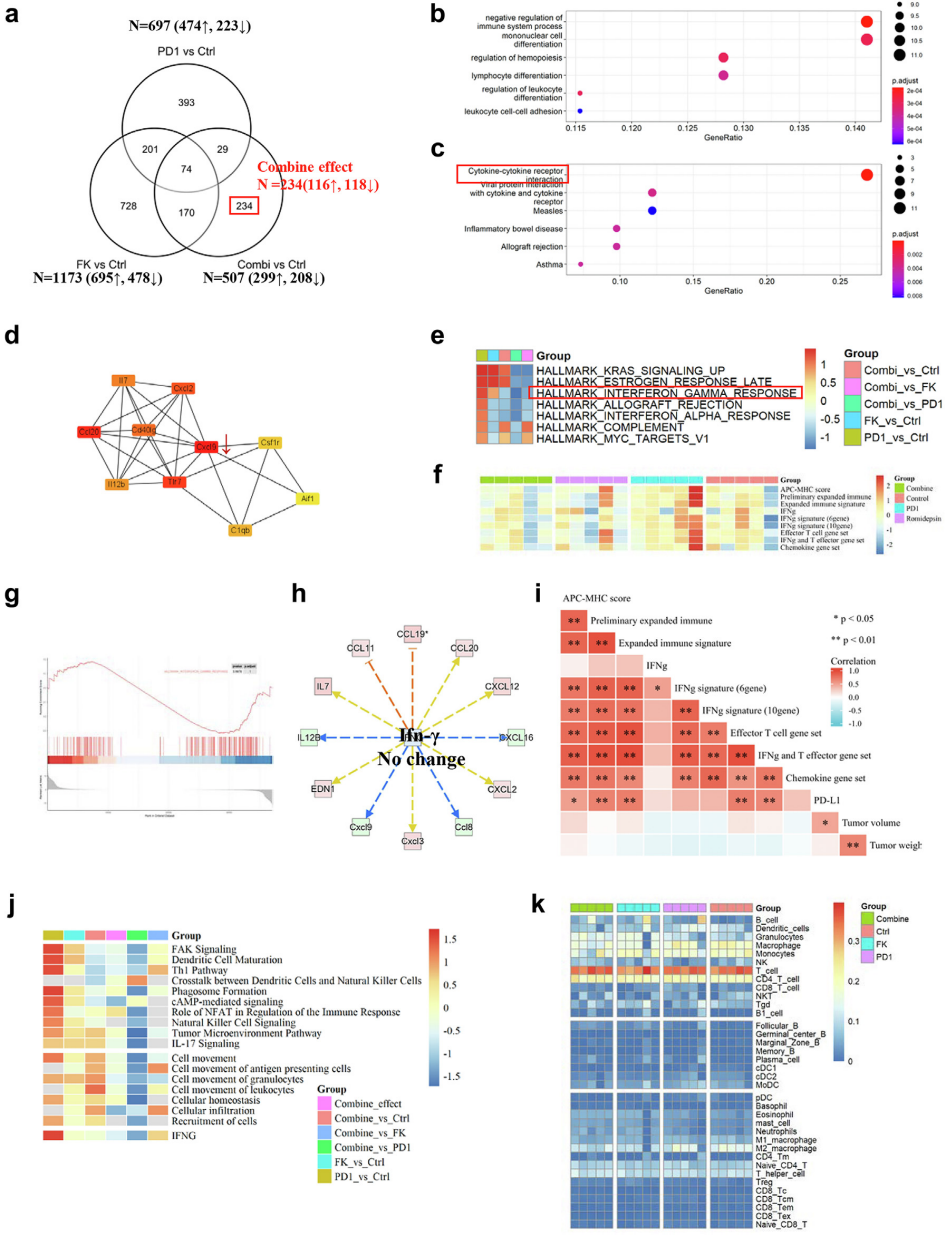
Combination of romidepsin and humanized PD1 antibody show no significant changes in T-cell chemokine expression and IFN-γ response. **a**, Summary of shared and nonoverlapping DEGs with several upregulated and downregulated genes across treatment groups is shown. 234 genes which only regulated in combination groups are definite as the combined effect. **b**, Go analysis of downregulated genes in combined effect. **c**, KEGG analysis of downregulated (Below) genes in combined effect. **d**, String protein–protein analysis of combined effect to explore hub genes in the regulated network. **e**, Heatmap of GSEA analysis in different comparisons. **f**, Heatmap of gene set expression scores in different groups. **g**. GSEA analysis of IFN-γ response in anti-PD1/romidepsin. **h**, downstream genes which may be regulated by IFN-γ. **i**, the correlation analysis of each score and tumour volume or weight. **j**, Ingenuity pathway analysis of canonical pathway (Upper) and diseases & function (Below) changes in each comparison. **k**, Immune cell proportion estimated based on RNA-seq data (∗*P*＜0.05，∗∗*P*＜0.001, Spearman correlation analysis).

To understand why romidepsin cannot synergize with the PD1 antibody, we also performed RNA-seq of tumour tissues to illustrate the underlying mechanism. A single agent PD1 antibody or romidepsin modulated the expression of a similar number of genes (697 and 1173 DEG, respectively). However, the combination only yielded a few changes, and 234 (234/507) non-overlapping DEG, most of which were downregulated, were uniquely modulated by the combination treatment (Fig. 4a, Supplementary Table S3). GO/KEGG cluster analysis of DEG in the combination group showed that combination treatment significantly downregulated genes involved in immune system processes, such as cytokine–cytokine receptor interaction (Fig. 4 b-c). To investigate the hub genes in the combination group, string protein–protein interaction network analysis suggested that the hub genes are Cxcl9, Ccl20, Tlr7, and Cxcl2 (Fig. 2d). However, the expression of Cxcl9 was downregulated in the combination group compared to PD1 monotherapy group. IPA upstream analysis suggested that IFN-γ is an upstream regulator with no significant changes and cannot regulate several downstream genes (Fig. 4h, Supplementary Table S4).

GSEA hallmark analysis showed that interferon alpha and gamma response hallmarks were significantly downregulated in the combination group compared to those in the monotherapy group (Fig. 4e, g). IPA comparison analysis in canonical pathways suggests that immune pathways, such as dendritic cell maturation, tumour microenvironment pathway, natural killer cell signalling, and the role of NFAT in the regulation of the immune response, are all inhibited in the combination therapy (Fig. 4j). IPA comparison analysis of diseases and biological functions also suggested that immune cell recruitment, movement, homeostasis, and migration were inhibited by the combination treatment (Fig. 4j). ImmuneCellAI-mouse estimated the abundance of immune cells, indicating no significant changes with the combination (Fig. 4k, Supplementary Fig. S4l). Gene set scores in the combination treatment were not significantly over-expressed (Fig. 4f, Supplementary Fig. S4a–k). Tumour volume and weight were not significantly correlated with any of the scores (Fig. 4f).

Taken together, these data reveal that the romidepsin/anti-PD1 combination shows no synergistic in the process of immune cell migration, infiltration, and killing of cancer cells.

### Ifn-γ is an essential response factor in the anti-PD1/chidamide combination, but not in the anti-PD1/romidepsin combination

We suggested Ifn-γ is an essential regulator for the combination in the previous work. Then, we use anti-Ifn-γ to validate its importance in vivo. Although the previous article suggested that the most efficient dosage of romidepsin in vivo is 0.3 mg/kg, we also tesedt the anti-PD1/romidepsin combination in a higher dose of 1.5 mg/kg. Ifn-γ antagonist dosing schedule is shown in Fig. 2f. The tumour growth curve showed that the high dose or low dose anti-PD1/chidamide were both superior to high dose or low dose anti-PD1/romidepsin. There is no difference between low-dose and high-dose anti-PD1/romidepsin. When using Ifn-γ antibody to neutralize Ifn-γ secretion, the efficacy of the combination group was also reduced accordingly. Tumour weight changes show the same trends. Changes in mouse weight in each group were not obvious (Fig. 2g–j) but one mouse in the high dose anti-PD1/romidepsin died on day 12.

**Fig. 5:**
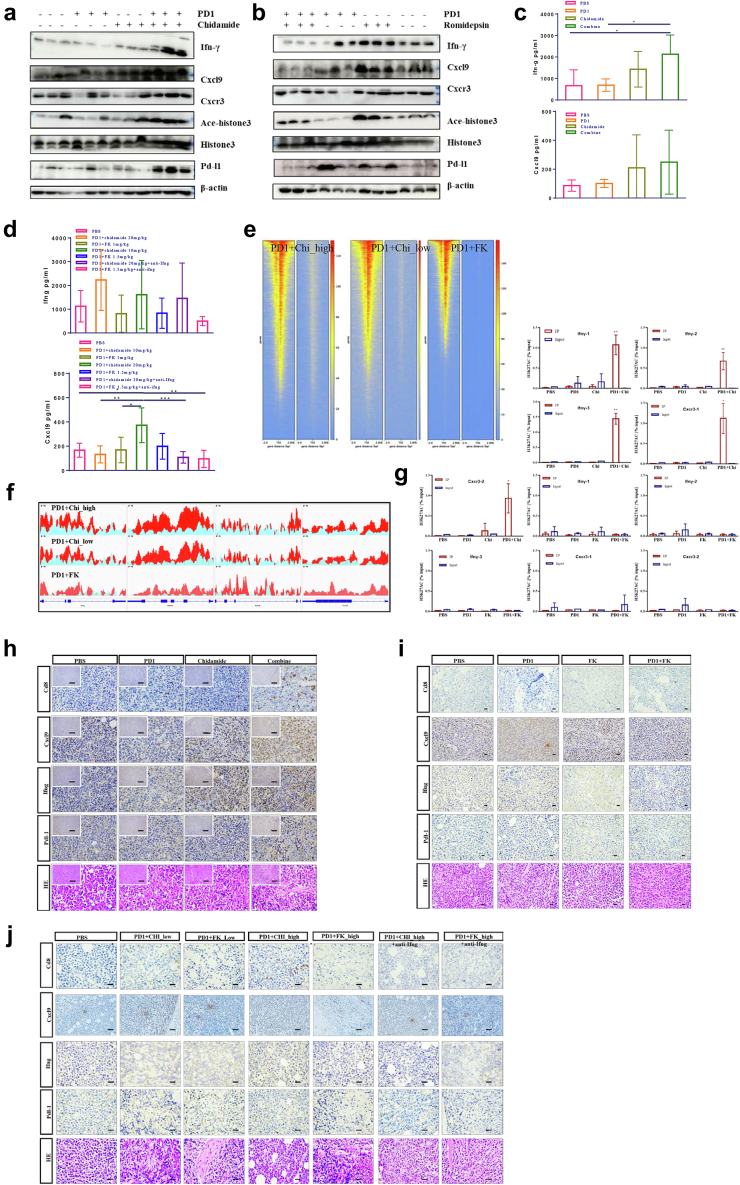
Anti-PD1/chidamide shows an immune response with epigenetic regulation. **a,** Western blot of Ifn-γ, Cxcl9, Cxcr3, Acetylated histone H3, histone H3, Pd-l1, and β-actin in anti-PD1/chidamide and each monotherapy. **b,** Western blot of Ifn-γ, Cxcl9, Cxcr3, Acetylated histone H3, histone H3, Pd-l1, and β-actin in anti-PD1/romidepsin and each monotherapy. **c**, ELISA of tumour lysis in anti-PD1/chidamide. **d**, ELISA of tumour lysis in anti-IFN-γ neutralization. **e**, Heatmap of ChIP-seq in high-dose, low-dose anti-PD1/chidamide, high-dose anti-PD1/romidepsin. **f**, Acetylated level of immune responses factor Ifn-γ, Gzmb, Gzmk, and Cxcr3 (left to right). **g**, ChIP-qPCR validation of Ifn-γ and Cxcr3 in different groups of anti-PD1/chidamide and anti-PD1/romidepsin. **h**, immunohistochemistry staining of Cd8, Cxcl9, Ifn-γ, and Pd-l1 in anti-PD1/chidamide. i, immunohistochemistry staining of Cd8, Cxcl9, Ifn-γ, and Pd-l1 in anti-PD1/romidepsin. **j**, immunohistochemistry staining of Cd8, Cxcl9, Ifn-γ, and Pd-l1 in anti-PD1/romidepsin or anti-PD1/chidamide ± anti- Ifn-γ (∗*P*＜0.05，∗∗*P*＜0.001, ∗∗∗*P*＜0.0001, Mann–Whitney U-test and one-way ANOVA).

To validate the immune gene changes discovered from RNA-seq at the protein level, we performed western blotting in each group of tumours. We found that Ifn-γ, Cxcl9, Cxcr3, acetylated histone H3, and Pd-l1 protein are all increased in the anti-PD1/chidamide compared to monotherapy (Fig. 5a), whereas no significant changes can observe in the anti-PD1/romidepsin (Fig. 5b). The Ifn-γ and Cxcl9 secretion in tumours were increased in the anti-PD1/chidamide and were somewhat reduced in the anti-Ifn-γ detected by ELISA (Fig. 5c and d). In situ staining, tissue slides showed higher Cd8 infiltration and Cxcl9, Ifn-γ, Pd-l1 expression in the anti-PD1/chidamide, but not show in the anti-PD1/romidepsin (Fig. 5h–j).

HDAC inhibitor is involved in the epigenetic regulation of tumour progression. We used ChIP-Seq to compare epigenetic changes in the high-dose, low-dose anti-PD1/chidamide, and high-dose anti-PD1/romidepsin groups. ChIP-seq reveal that more genes in the anti-PD1/chidamide combination group were acetylated near the transcription start site, thereby activating the transcriptional expression of immune-related effectors (Fig. 5e). The effector acetylation level increased more significantly in the anti-PD1/chidamide combination group (Fig. 5f). According, H3K27 acetylated level (% input) in the PD1/chidamide combination was also higher at several regions in Ifn-γ and Cxcr3 compared to each monotherapy, but with no obvious acetylated in PD1/romidepsin combination and each monotherapy (Fig. 5g).

### PD1 antibody combined with chidamide shows promising efficacy in the clinic

**Fig. 6:**
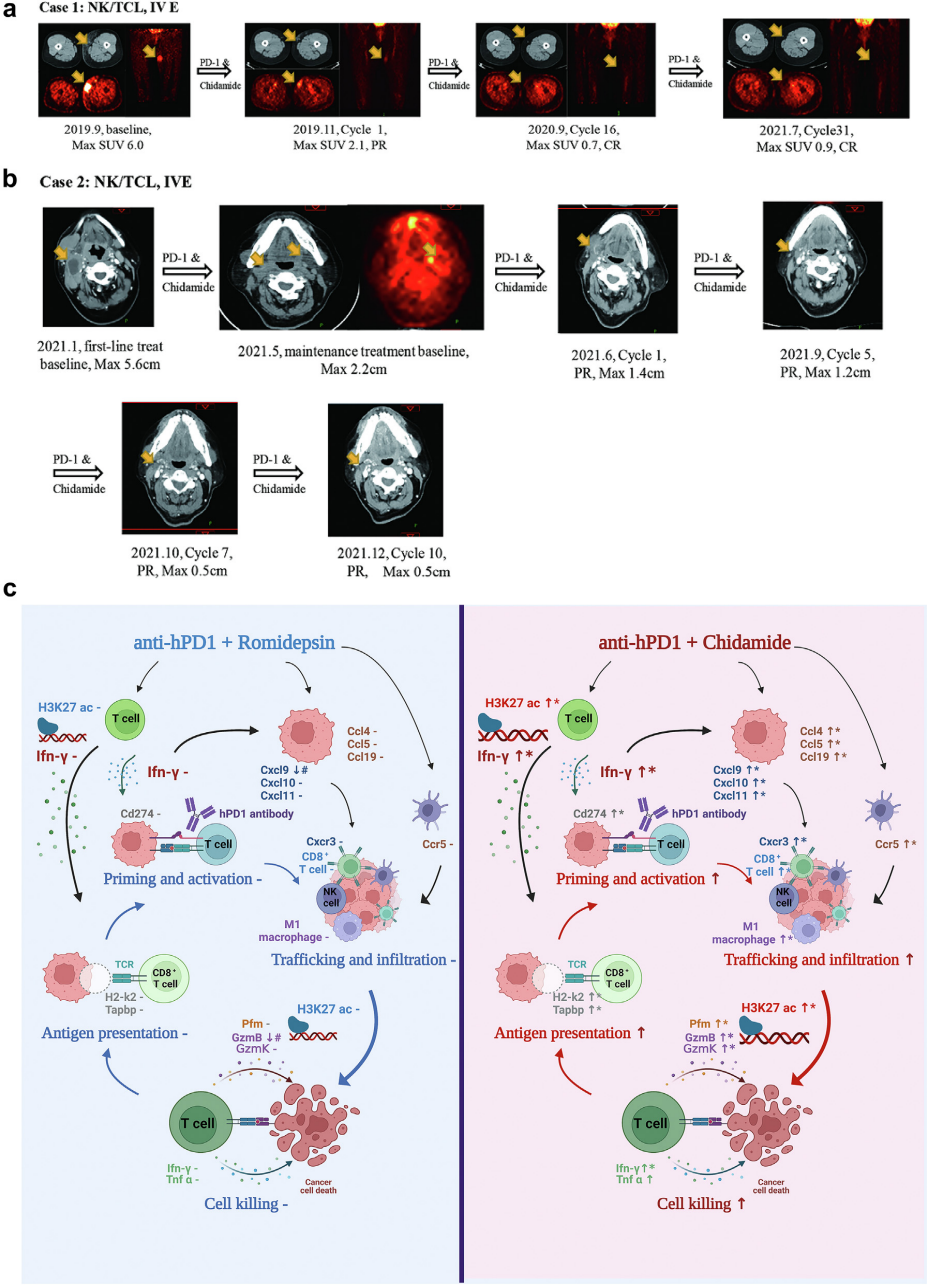
Anti-PD1/chidamide shows promising efficiency in clinical and its mechanism scenario. **a**, Case 1 is a female patient with NK-T cell lymphoma who received more than 2-year treatment of anti-PD1 and chidamide after refractory from first-line chemotherapy. **b**, Case 2 is a female patient with NK-T cell lymphoma who received anti-PD1 and chidamide as maintenance treatment after first-line chemotherapy finished. **c**, anti-PD1/Chidamide combination induces chemokine expression in multiple cell types resulting in enhanced immune cell recruitment, trafficking and infiltration. Chemokine ligands and receptors binding include Cxcr3-Cxcl9/10/11 in CD8 T-tumour cells or NK-tumour cells and Ccr5-Ccl4/Ccl5/Ccl20 in dendritic-tumour cells. Besides, the combination enhances IFN-γ expression and acetylated level in multiple cell types resulting in enhanced antigen presentation, T cell activation, immune cell infiltration, and tumour cell killing. Cd274 is also upregulated by IFN-γ as a feedback loop and tumour cells become more responsive to anti-PD1 treatment. By contrast, there was no additive anti-tumour effect when romidepsin combined with anti-PD1, including no changes in chemokines and IFN-γ responses.

In the clinical setting, we retrospectively collected data from two patients treated with PD1 antibody and chidamide. Case 1 involved a 59-year-old woman. Disease relapse occurred after two months of first-line treatment with multiple extensive lesions (Table 1). Then, nivolumab combined with chidamide was treated under the patient's informed consent. Surprisingly, the tumour shrank rapidly after two cycles of the chemo-free regimen (Fig. 6a). The patient was well tolerated, not as toxic as the first-line regimen, and combination therapy has been continued to date. The patient is still alive in March 2022. Case 2 was a 56-year-old woman. Although the patient showed a partial response after six cycles of first-line chemotherapy with severe hematological toxicity, extensive lesions with a maximum diameter of 2.2 cm were still challenging. Therefore, our multidisciplinary team suggests that patients continue to maintain treatment. After receiving ten cycles of PD1 antibody and chidamide, the max diameter of the tumour shrunk to less than 0.5 cm (Fig. 6b).

**Table 1:** Clinical characteristics of two NK-T cell lymphoma patients who were treated with PD1 antibody and HDAC inhibitor.

Cases	Sex	Age	Stage	ECOG	Pathology	PINK/PINK-E	Sites	Primary treatment	PD1 treatment	PFS	OS	Survival
1	Female	59	IV	1	NKTCL, CD3+, CD5-, CD8+, CD56-, CD30 20%, EBER+, Ki-67 50%, Granzyme B +, EBER+	3/3, EBV DNA 0 copies/ml	right shoulder, both upper limbs, right buttocks, and subcutaneous of both lower limbs; invasion of the right upper and middle turbinate; invasion of both necks, right axilla, mediastinum and double hilum, retroperitoneum, left iliac vessels, bilateral lateral inguinal lymph nodes	GELOX∗6,PD, PFS 2 months; Grade 3 hematological toxicity, grade 2 abnormal liver function, grade 3 hypothyroidism	Nivolumab 200 mg d1+Chidamide 20 mg po d3,6,9,12/Q3w; AE: Grade 1 hematological toxicity; Grade 1 fatigue	30 months+	36 months+	Yes
2	Female	56	IV	1	NKTCL, CD3+, CD5-, CD56+, CD30-, EBER+, Ki-67 90%, PD-L1 22C3 CPS = 1	3/4, EBV 4.48 copies/ml	Ethmoid sinus, nasopharynx, oropharynx, left orbit; bilateral zone Ib/II, zone III lymph nodes	l-asparaginase + GDP∗6, PR; Grade 3 hematological toxicity; Deauville 4	Maintenance treatment, Sintilimab 200 mg d1+ Chidamide 20 mg po d3,6,9,12/Q3w; AE: Grade 1 rash	10 months+	16 months + a	Yes

## Discussion

Since the approval of immunotherapy in 2011, it has completely reformed the treatment pattern of tumours, either in solid tumours or hematological malignancies. However, it has to be admitted that the efficacy of monotherapy is limited. In 2022, Samik et al. reported that 4062 of 4897 active trials (83%) tested PD1/PDL1 combination therapies with other immuno-oncology therapies, targeted therapies, chemotherapies, and radiotherapies.^27^ Unfortunately, the failure rate of immunotherapy combinations remains a challenge, even in the clinical trial phase 2 to phase 3 transition. Only 12.4% of immunotherapy could be successful from Phase 1 to biological license applications, and the application of biomarkers to select potential benefits patients may double the success rate (15.9% vs. 7.6%), which is particularly important in Phase 2 clinical trial transition (46.3% vs. 28.3%).^28^ Failure Phase 3 head-to-head randomized trials included but were not limited to KEYLYNK-010 (pembrolizumab plus PARP inhibitor, prostate cancer), LAEP-007 (pembrolizumab plus lenvatinib, lung cancer), KEYNOTE-604 (pembrolizumab plus chemotherapy, lung cancer), KEYNOTE-062 (pembrolizumab plus chemotherapy, gastric cancer), Imblaze-370 (atezolizumab plus combimetinib, colon cancer), and IMPASSION-131 (atezolizumab plus chemotherapy, breast cancer). Appropriate preclinical models for drug efficacy evaluation, efficient patient selection biomarkers, and rational clinical research design strategies are key to improving the success rate of clinical trials. It may cost a lot of labour forces and financial forces to launch a new Phase 3 randomized clinical trial, thus this study aims to test the efficacy of the combination of anti-PD1 and HDAC inhibitors in NKTCL immunocompetent preclinical modes, factor analysis of the contribution of each agent, and explore potential response biomarkers.

Previous preclinical studies have reported that HDAC inhibitors may augment the response to immunotherapy in preclinical models of solid tumours, but there are no pre-clinical reports on NKTCL. This study emphasizes the superiority of chidamide, rather than romidepsin, in immunotherapy combinations and provides preclinical evidence for the expansion of combination indications. Zheng et al. reported that romidepsin can induce tumour cells and T cells to secrete T cell chemokines and recruit T cells to accumulate, thus improving the efficacy of PD1 immunotherapy in a mouse lung adenocarcinoma model.^29^ Woods et al. reported that HDAC inhibitors upregulated PD-L1 expression in melanoma and augmented the response to PD1 antibody.^30^ In liver cancer, breast cancer, bladder cancer, renal clear cell cancer, and colorectal cancer, HDAC inhibitors have also been reported to upregulate the levels of tumour antigen/costimulatory molecules/receptors (such as major histocompatibility complex), activate NK cells or cytotoxic T cells, and relieve the inhibition of immunosuppressive cells (such as regulatory T cells and myeloid-derived suppressor cells).^26^^,^^31^, ^32^, ^33^, ^34^, ^35^, ^36^ Unfortunately, the combination of an HDAC inhibitor and anti-PD1 treatment in clinical trials has shown limited efficacy in solid tumours. Pembrolizumab plus vorinostat or entinostat only showed about 10% ORR in non-small cell lung cancer. Pembrolizumab or atezolizumab plus vorinostat showed 4–10% ORR in different types of breast cancer. Pembrolizumab plus vorinostat showed 32% ORR in head and neck squamous cell carcinoma.^37^ PEMDAC phase two clinical trial assessing entinostat with pembrolizumab in metastatic uveal melanoma reported an ORR of 14%.^38^

Because the mechanism of the PD1 antibody is complex and mainly depends on the immune system, it is difficult to mimic the immune process in vitro. Therefore, this study investigated the efficacy of these combinations in genetically engineered immunocompetent mouse models. The antigen-binding epitope of the antibody is a key factor in determining its efficacy. Different clones of murine PD1 antibody may result in different responses. The use of a commercial humanized PD1 monoclonal antibody to test the efficacy of the combination may be more direct and closer to clinical application. The extracellular domain of human PD1 is different from that of dogs, rats, and mice (dogs: 80% similar, 72% identical; rats: 76% similar, 66% identical; mice: 74% similar, 62% identical), but is similar to that of rhesus monkeys and cynomolgus monkeys (99% and 96%, respectively).^39^ Therefore, it was necessary to replace the sequence of mouse PD1 with human PD1 when evaluating the efficacy of humanized PD1 antibodies in mice. Considering the dose setting, the anti-tumour effect of PD1 antibody is mainly triggered by blocking PD-1 expression on T cells, rather than by direct binding to cancer cells. Once antibodies fully occupy the T cell receptor, the exposure–response relationship between different tumour types is expected to be similar.^40^ Therefore, the PD1 antibody was set as a fixed dose in this study, and dose escalation was expected with HDAC inhibitors. Sasakawa et al.^41^ reported that the optimal dosage of romidepsin in an immune deficiency mouse model was 0.32 mg/kg, twice a week, and the maximum tolerated dose was 1 mg/kg. Three doses of romidepsin were investigated in the present study but with limited efficiency. The statistical analysis of the combination index suggested that anti-PD1/chidamide is synergistic. Clinical cases suggest that the toxicity of this chemo-free regimen is milder than that of l-asparaginase-based chemotherapy, and chidamide is an oral regimen that may be a more convenient choice for patients.

Mechanistic analysis not only focuses on a single gene or protein but also investigates the changes in hallmark pathways. HDAC inhibitors are epigenetic agents that regulate histone acetylation, chromatin structure and gene transcription. Currently, HDAC inhibitors such as chidamide, belinostat, and vorinostat are accelerated approved for certain subtypes of T-cell lymphoma in some countries. The accelerated approval status for romidepsin for the treatment of patients with relapsed/refractory peripheral T-cell lymphoma has been withdrawn following the results of the confirmatory phase 3 Ro-CHOP trial in August 2021. Romidepsin and chidamide have different structures and HDAC inhibitory activities (Supplementary Fig. S1d).^42^^,^^43^ Although we didn't find any significantly different changes between romidepsin and chidamide monotherapy, immune responses involving antigen presentation, priming and activation, trafficking and infiltration, and cell killing are activated in anti-PD1/chidamide but no obvious changes in anti-PD1/romidepsin (Fig. 6c). In anti-PD1/chidamide, the hub genes were chemokines and Ifn-γ and the acetylated level of immune effect factors such as Ifn-γ, Granzyme B, and Granzyme K are increased, accompanied by more density CD8 T cell infiltration. The hallmark pathway in the Ifn-γ responses is highly activated in anti-PD1/chidamide but without significant changes in anti-PD1/romidepsin. Ifn-γ and chemokine-related genes may be the core mechanism of the combination, and the expression of these genes is correlated with tumour regression, so the Ifn-γ signature may be useful for the selection of benefits patients in clinical trials.

This study not only tested the combination efficacy in multiple tumour models but also retrospectively collected clinical cases, which may provide clinical evidence of potential benefits. The two R/R NKTCL cases showed promising results. The patients seemed more tolerant to this chemo-free regimen than other chemotherapy regimens, even in continuous applications for more than one year.

Some limitations still exist in our study: 1) Because immunotherapy heavily depends on the immune system, it is hard to test combination efficiency in patient-derived xenografts. 2) Although we tried three dosages in anti-PD1/romidepsin based on previous reports, it is hard to test every different dosage matrix in vivo.

In summary, combination therapy is an important means to improve anti-tumour efficacy and overcome drug resistance, but whether synergistic or additive effects among combined antitumor agents should be rationally verified. PD1 antibody combined with chidamide enhances T-cell chemokine expression and augments the IFN-γ response in preclinical NKTCL immunocompetent models. IFN-γ signatures may be good response biomarkers for the selection of potentially benefit patients. Moreover, the chemo-free regimen of chidamide with anti-PD1 treatment showed promising efficacy and mild toxicity in clinical cases.

## Contributors

Tingyu Wen, Yuankai Shi, Fei Ma, and Peng Liu conceived the project and designed the experiments. Tingyu Wen, Guangyi Sun, and Wenxin Jiang conducted experiments. Tingyu Wen and Guangyi Sun analyzed, discussed the results, and wrote the manuscript. Xiaohui He contributed one clinical case. Tingyu Wen revised the manuscript. Tingyu Wen and Peng Liu verified the underlying data. All authors read and approved the final manuscript.

## Data sharing statement

The data supporting the findings of this study are available from the corresponding author upon reasonable request.

## Declaration of interests

The authors declare no potential conflicts of interest.

## Appendix A Supplementary data

Supplementary Fig. S4

Westernblot

## References

[1] Sun J., Yang Q., Lu Z. (2012). Distribution of lymphoid neoplasms in China: analysis of 4,638 cases according to the World Health Organization classification. Am J Clin Pathol.

[2] Haverkos B.M., Pan Z., Gru A.A. (2016). Extranodal NK/T cell lymphoma, Nasal Type (ENKTL-NT): an update on epidemiology, clinical presentation, and Natural History in North American and European cases. Curr Hematol Malig Rep.

[3] Kwong Y.L., Kim W.S., Lim S.T. (2012). SMILE for natural killer/T-cell lymphoma: analysis of safety and efficacy from the Asia Lymphoma Study Group. Blood.

[4] Yang Y., Wang Y., Liu X. (2020). Progression-free survival at 24 months and subsequent survival of patients with extranodal NK/T-cell lymphoma: a China Lymphoma Collaborative Group (CLCG) study. Leukemia.

[5] Panjwani P.K., Charu V., DeLisser M., Molina-Kirsch H., Natkunam Y., Zhao S. (2018). Programmed death-1 ligands PD-L1 and PD-L2 show distinctive and restricted patterns of expression in lymphoma subtypes. Hum Pathol.

[6] Kwong Y.L., Chan T., Tan D. (2017). PD1 blockade with pembrolizumab is highly effective in relapsed or refractory NK/T-cell lymphoma failing l-asparaginase. Blood.

[7] Li J., Tao R., Fan L. (2020). Sintilimab for relapsed/refractory (r/r) extranodal NK/T cell lymphoma (ENKTL): extended follow-up on the multicenter, single-arm phase II trail (ORIENT-4). J Clin Oncol.

[8] Tao R., Fan L., Song Y. (2021). Sintilimab for relapsed/refractory extranodal NK/T cell lymphoma: a multicenter, single-arm, phase 2 trial (ORIENT-4). Signal Transduct Target Ther.

[9] Kim S.J., Lim J.Q., Laurensia Y. (2020). Avelumab for the treatment of relapsed or refractory extranodal NK/T-cell lymphoma: an open-label phase 2 study. Blood.

[10] Shi Y., Dong M., Hong X. (2015). Results from a multicenter, open-label, pivotal phase II study of chidamide in relapsed or refractory peripheral T-cell lymphoma. Ann Oncol.

[11] Foss F., Advani R., Duvic M. (2015). A Phase II trial of Belinostat (PXD101) in patients with relapsed or refractory peripheral or cutaneous T-cell lymphoma. Br J Haematol.

[12] Coiffier B., Pro B., Prince H.M. (2012). Results from a pivotal, open-label, phase II study of romidepsin in relapsed or refractory peripheral T-cell lymphoma after prior systemic therapy. J Clin Oncol.

[13] Karagiannis D., Rampias T. (2021). HDAC inhibitors: dissecting mechanisms of action to counter tumor heterogeneity. Cancers.

[14] Zhang H., Deng M., Lin P. (2018). Frontiers and opportunities: highlights of the 2(nd) annual conference of the Chinese antibody society. Antib Ther.

[15] Patel G.V., Masucci M.G., Winberg G., Klein G. (1988). Expression of the Epstein-Barr virus encoded EBNA-1 gene in stably transfected human and murine cell lines. Int J Cancer.

[16] Gays F., Unnikrishnan M., Shrestha S. (2000). The mouse tumor cell lines EL4 and RMA display mosaic expression of NK-related and certain other surface molecules and appear to have a common origin. J Immunol.

[17] Zhao Y., Harrison D.L., Song Y., Ji J., Huang J., Hui E. (2018). Antigen-presenting cell-Intrinsic PD-1 neutralizes PD-L1 in cis to attenuate PD-1 signaling in T cells. Cell Rep.

[18] BLISS C.I. (1939). The toxicity OF POISONS applied JOINTLY1. Ann Appl Biol.

[19] Schaer D.A., Geeganage S., Amaladas N. (2019). The folate pathway inhibitor pemetrexed pleiotropically enhances effects of cancer immunotherapy. Clin Cancer Res.

[20] Love M.I., Huber W., Anders S. (2014). Moderated estimation of fold change and dispersion for RNA-seq data with DESeq2. Genome Biol.

[21] Subramanian A., Tamayo P., Mootha V.K. (2005). Gene set enrichment analysis: a knowledge-based approach for interpreting genome-wide expression profiles. Proc Natl Acad Sci U S A.

[22] Kashyap A.S., Schmittnaegel M., Rigamonti N. (2020). Optimized antiangiogenic reprogramming of the tumor microenvironment potentiates CD40 immunotherapy. Proc Natl Acad Sci U S A.

[23] Szklarczyk D., Gable A.L., Nastou K.C. (2021). The STRING database in 2021: customizable protein-protein networks, and functional characterization of user-uploaded gene/measurement sets. Nucleic Acids Res.

[24] Miao Y.R., Xia M., Luo M., Luo T., Yang M., Guo A.Y. (2021). ImmuCellAI-mouse: a tool for comprehensive prediction of mouse immune cell abundance and immune microenvironment depiction. Bioinformatics.

[25] Ayers M., Lunceford J., Nebozhyn M. (2017). IFN-γ–related mRNA profile predicts clinical response to PD-1 blockade. J Clin Invest.

[26] Bretz A.C., Parnitzke U., Kronthaler K. (2019). Domatinostat favors the immunotherapy response by modulating the tumor immune microenvironment (TIME). J Immunother Cancer.

[27] Upadhaya S., Neftelinov S.T., Hodge J., Campbell J. (2022). Challenges and opportunities in the PD1/PDL1 inhibitor clinical trial landscape. Nat Rev Drug Discov.

[28] Brown D.G., Wobst H.J., Kapoor A., Kenna L.A., Southall N. (2021). Clinical development times for innovative drugs. Nat Rev Drug Discov.

[29] Zheng H., Zhao W., Yan C. (2016). HDAC inhibitors enhance T-cell chemokine expression and augment response to PD-1 immunotherapy in lung adenocarcinoma. Clin Cancer Res.

[30] Woods D.M., Sodre A.L., Villagra A., Sarnaik A., Sotomayor E.M., Weber J. (2015). HDAC inhibition upregulates PD-1 ligands in melanoma and augments immunotherapy with PD-1 blockade. Cancer Immunol Res.

[31] Llopiz D., Ruiz M., Villanueva L. (2019). Enhanced anti-tumor efficacy of checkpoint inhibitors in combination with the histone deacetylase inhibitor Belinostat in a murine hepatocellular carcinoma model. Cancer Immunol Immunother.

[32] Orillion A., Hashimoto A., Damayanti N. (2017). Entinostat neutralizes myeloid-derived suppressor cells and enhances the antitumor effect of PD-1 inhibition in murine models of lung and renal cell carcinoma. Clin Cancer Res.

[33] Booth L., Roberts J.L., West C., Von Hoff D., Dent P. (2020). GZ17-6.02 initiates DNA damage causing autophagosome-dependent HDAC degradation resulting in enhanced anti-PD1 checkpoint inhibitory antibody efficacy. J Cell Physiol.

[34] McCaw T.R., Li M., Starenki D. (2019). Histone deacetylase inhibition promotes intratumoral CD8+ T-cell responses, sensitizing murine breast tumors to anti-PD1. Cancer Immunol Immunother.

[35] Burke B., Eden C., Perez C. (2020). Inhibition of histone Deacetylase (HDAC) enhances checkpoint blockade efficacy by rendering bladder cancer cells visible for T cell-mediated destruction. Front Oncol.

[36] Noonepalle S., Shen S., Ptacek J. (2020). Rational design of suprastat: a novel selective histone deacetylase 6 inhibitor with the ability to potentiate immunotherapy in melanoma models. J Med Chem.

[37] Borcoman E., Kamal M., Marret G., Dupain C., Castel-Ajgal Z., Le Tourneau C. (2021). HDAC inhibition to prime immune checkpoint inhibitors. Cancers.

[38] Ny L., Jespersen H., Karlsson J. (2021). The PEMDAC phase 2 study of pembrolizumab and entinostat in patients with metastatic uveal melanoma. Nat Commun.

[39] Wang J., Fei K., Jing H. (2019). Durable blockade of PD-1 signaling links preclinical efficacy of sintilimab to its clinical benefit. MAbs.

[40] Freshwater T., Kondic A., Ahamadi M. (2017). Evaluation of dosing strategy for pembrolizumab for oncology indications. J Immunother Cancer.

[41] Sasakawa Y., Naoe Y., Inoue T. (2002). Effects of FK228, a novel histone deacetylase inhibitor, on human lymphoma U-937 cells in vitro and in vivo. Biochem Pharmacol.

[42] Ning Z.Q., Li Z.B., Newman M.J. (2012). Chidamide (CS055/HBI-8000): a new histone deacetylase inhibitor of the benzamide class with antitumor activity and the ability to enhance immune cell-mediated tumor cell cytotoxicity. Cancer Chemother Pharmacol.

[43] Yao Y., Tu Z., Liao C. (2015). Discovery of novel class I histone deacetylase inhibitors with promising in vitro and in vivo antitumor activities. J Med Chem.

