# Supplementary material for: Histone deacetylases inhibitor chidamide synergizes with humanized PD1 antibody to enhance T-cell chemokine expression and augment Ifn-γ response in NK-T cell lymphoma
Source: eBioMedicine. 2022 Dec 31;87:104420. doi: 10.1016/j.ebiom.2022.104420 (PMC9823149; doi:10.1016/j.ebiom.2022.104420)
Supplement: Supplementary Fig. S1 [file mmc5.pdf]

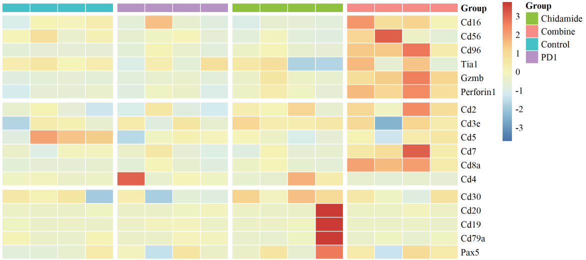

a

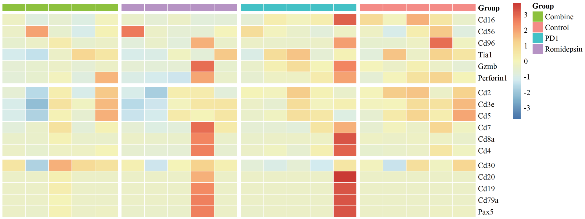

b

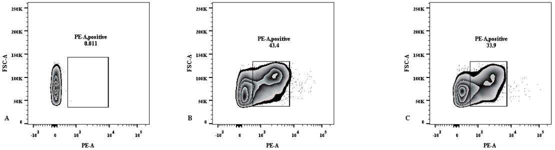

c

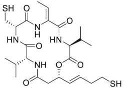

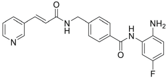

Romidepsin (FK228, 4) as its active reduced form
IC_{50} (HDAC1) = 0.1 nM
IC_{50} (HDAC2) = 1.4 nM
IC_{50} (HDAC3) = 4.8 nM
IC_{50} (HDAC4) = 9,570 nM
IC_{50} (HDAC5) > 10,000 nM
IC_{50} (HDAC6) = 149 nM
IC_{50} (HDAC7) > 10,000 nM
IC_{50} (HDAC8) = 25 nM
IC_{50} (HDAC9) > 10,000 nM
IC_{50} (HDAC10) = 39 nM
IC_{50} (HDAC11) = 2,390 nM

Chidamide (CS055/HBI-8000, 5)
IC_{50} (HDAC1) = 95 nM
IC_{50} (HDAC2) = 160 nM
IC_{50} (HDAC3) = 67 nM
IC_{50} (HDAC4) > 30,000 nM
IC_{50} (HDAC5) > 30,000 nM
IC_{50} (HDAC6) > 30,000 nM
IC_{50} (HDAC7) > 30,000 nM
IC_{50} (HDAC8) = 733 nM
IC_{50} (HDAC9) > 30,000 nM
IC_{50} (HDAC10) = 78 nM
IC_{50} (HDAC11) = 432 nM

d
