# Supplementary figures and images for: Histone deacetylases inhibitor chidamide synergizes with humanized PD1 antibody to enhance T-cell chemokine expression and augment Ifn-γ response in NK-T cell lymphoma
Source: eBioMedicine. 2022 Dec 31;87:104420. doi: 10.1016/j.ebiom.2022.104420 (PMC9823149; doi:10.1016/j.ebiom.2022.104420)

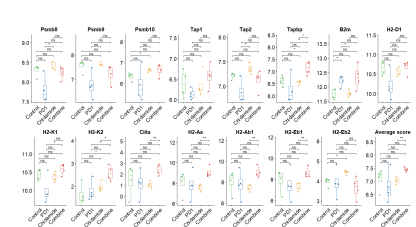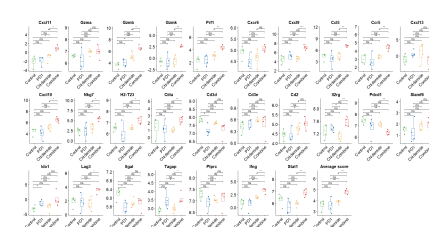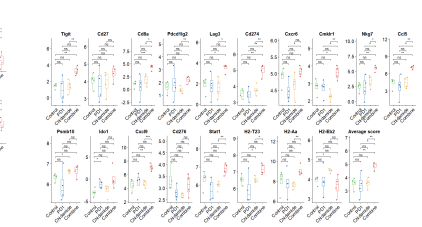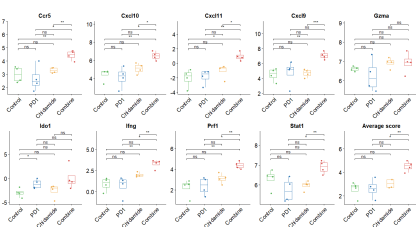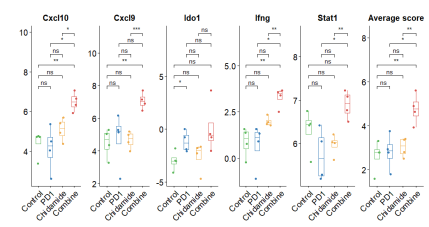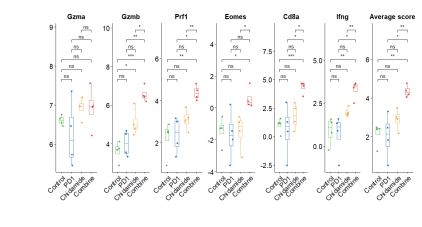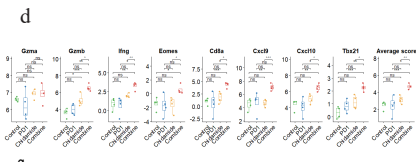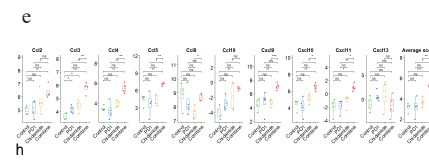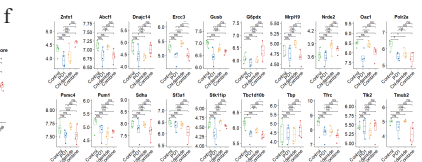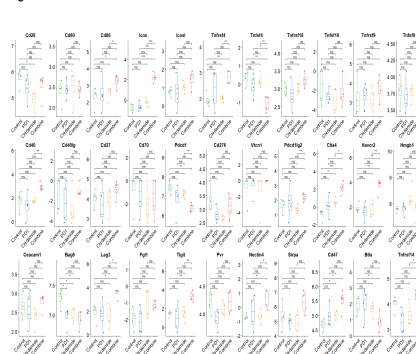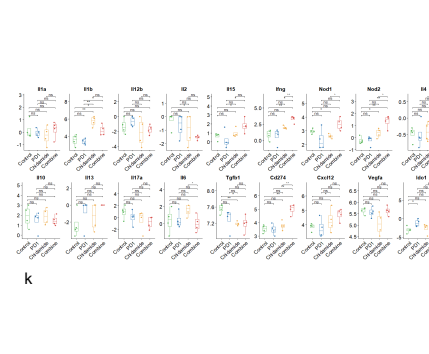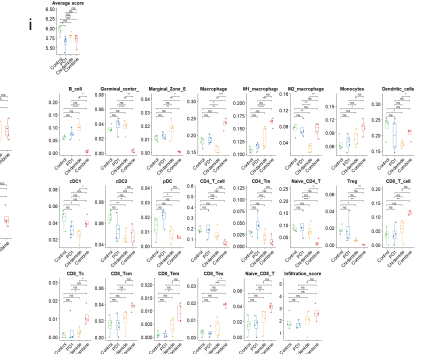

**j**

**k**

Supplement: Supplementary Fig. S3 [file mmc6.pdf]

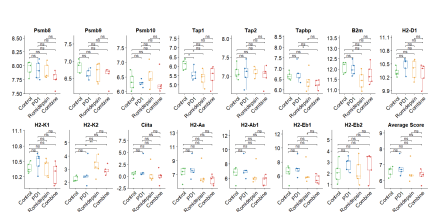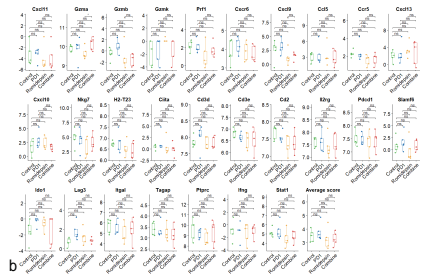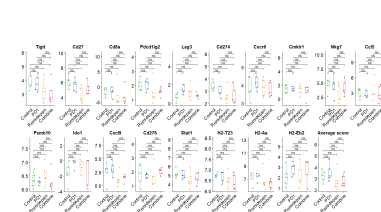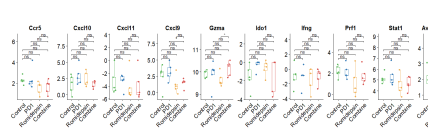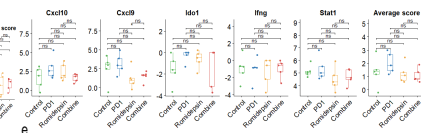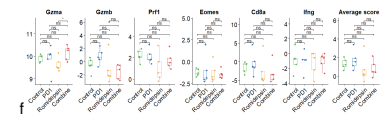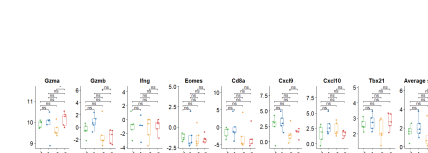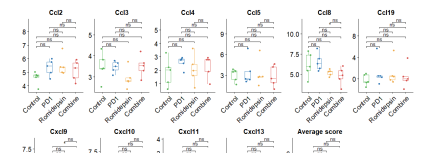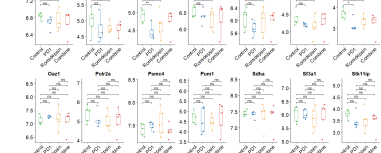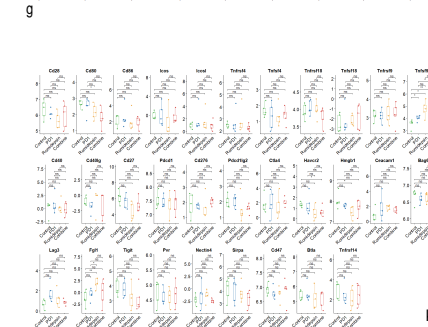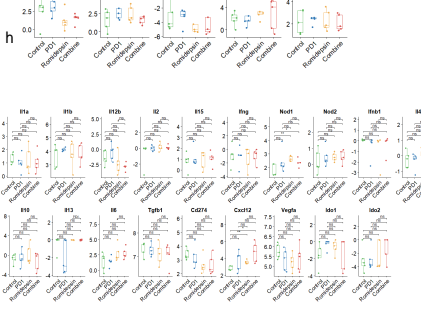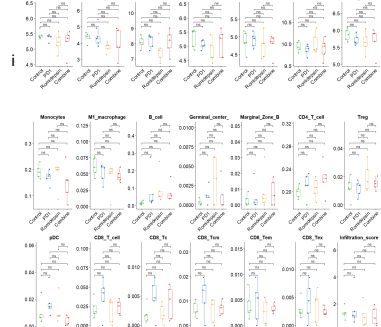

Supplement: Supplementary Fig. S4 [file mmc7.pdf]

ifng

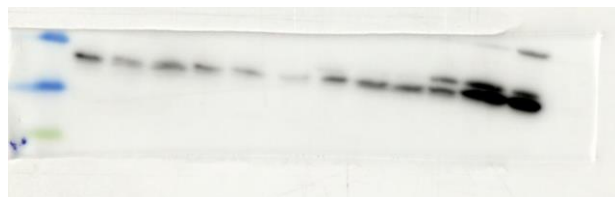

pdl1

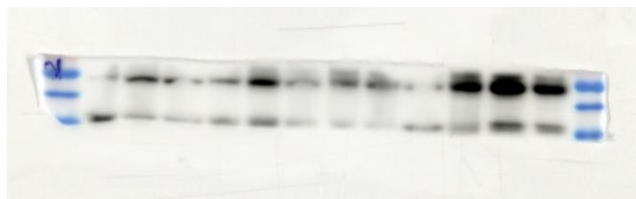

actin

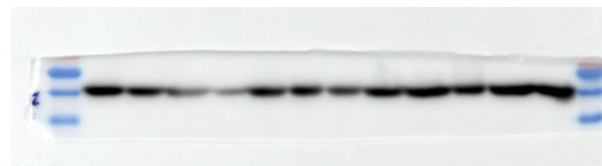

cxcr3

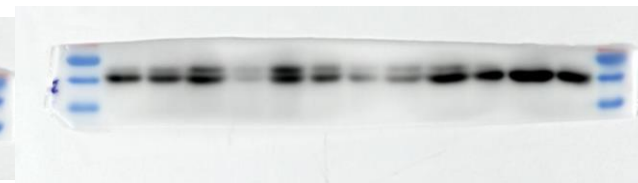

Ace-histone3

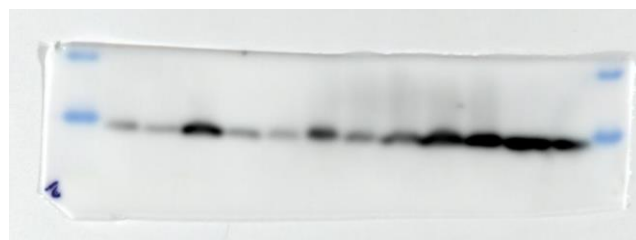

histone3

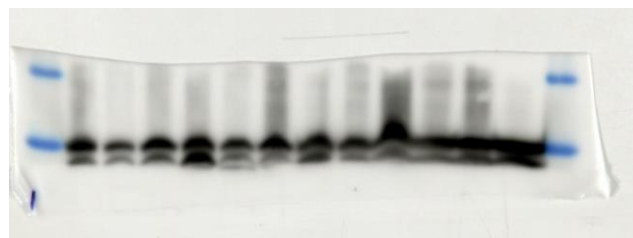

cxcl9

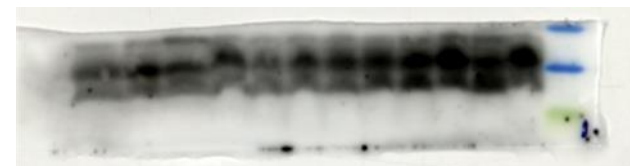

cxcr3

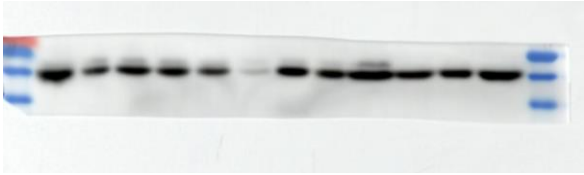

pdl1

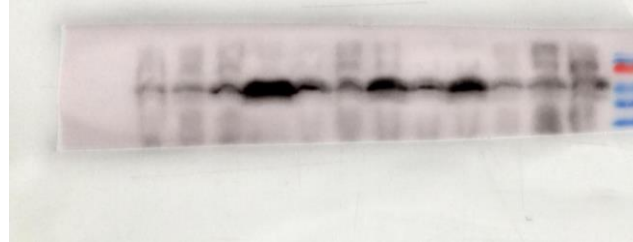

ifng

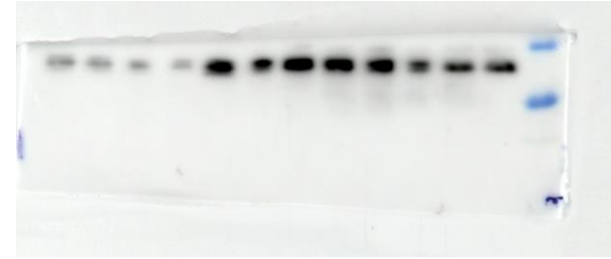

cxcl9

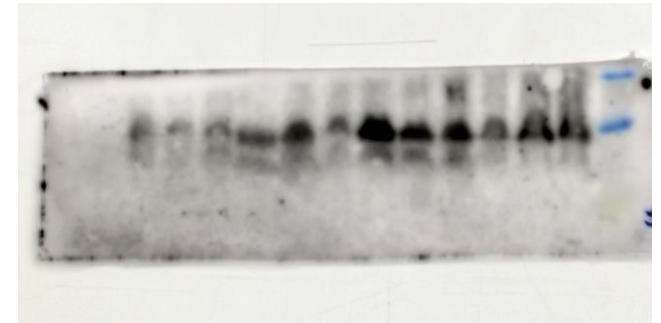

Ace-histone3

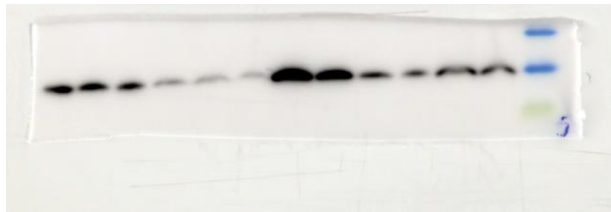

actin

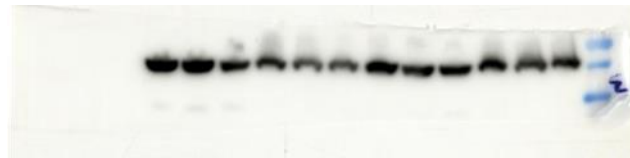

histone3

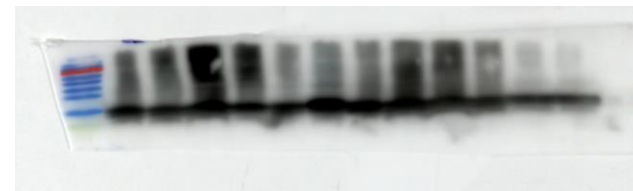

Supplement: Westernblot [file mmc8.pdf]
